# Gender disparities in delayed angina diagnosis: insights from 2001–2020 NHANES data

**DOI:** 10.1186/s12889-025-22214-4

**Published:** 2025-03-29

**Authors:** Naydeen Mostafa, Ahmed Sayed, Marwan Hamed, Muhiddin Dervis, Omar Almaadawy, Omar Baqal

**Affiliations:** 1 Faculty of Medicine, Ain Shams University, Cairo, Egypt; 2 Houston Methodist DeBakey Heart & Vascular Center, Houston, TX USA; 3 Department of Cardiovascular Medicine, Mayo Clinic College of Medicine, Rochester, MN USA; 4 Faculty of Medicine, Ankara Yilidirim Beyazit University, Ankara, Turkey; 5 Department of Internal Medicine, MedStar Health, Baltimore, MD USA; 6 Department of Cardiovascular Medicine, Mayo Clinic, Phoenix, AZ USA

**Keywords:** Angina, NHANES, Women’s health, Coronary artery disease, Gender disparity, Diagnostic delays

## Abstract

**Background:**

Women with coronary artery disease (CAD) are more likely than men to experience a delay in diagnosis, which is attributed to differences in clinical presentation. The objective of this study is to examine any persistent disparities in timely CAD diagnosis in the United States (U.S.) among women who present with clinically similar symptoms and demographic characteristics to their male counterparts.

**Methods:**

From the 2001 – 2020 National Health and Nutrition Examination Survey (NHANES) data, participants were categorized as having missed angina if they experienced angina and did not self-report a prior diagnosis of angina pectoris or CAD. We assessed the association between gender and missed angina using weighted multivariate logistic regression models representative of the U.S. population. Mortality follow-up data were available for participants up to December 31, 2018.

**Results:**

Of 874 participants with missed angina, 551 (63%) were women and 323 (37.0%) were men. Baseline characteristics showed that women and men with missed angina were more likely than their diagnosed counterparts to be younger, of ethnic minorities, uninsured, and smokers. Women with missed angina were more likely to be in a relationship than diagnosed women, while the opposite pattern was observed in men. The odds ratio of missed angina in women compared to men was 2.61 (95% CI: 1.73, 3.94) after adjusting for age, race, education, body mass index, smoking, alcohol use, income, insurance, and comorbidities. Among participants who had a cardiac cause of death, the odds of missed angina in women compared to men was 3.02 (95% CI: 1.18, 7.75) in the adjusted model.

**Conclusion:**

Women with similar CAD symptoms to their male counterparts still have higher odds of going undiagnosed. This relationship extends to individuals who ultimately die of cardiac causes. Potential solutions to this disparity include addressing overgeneralized perceptions of differences in the prevalence and presentation of CAD between genders and exploring targeted screening programs for women with risk factors. Further research accounting for healthcare access and proximity to care is needed to support our findings. Timely recognition of CAD in women is essential to decreasing preventable mortality.

## Introduction

Coronary artery disease (CAD) is the leading cause of death in the United States (U.S.), with a recent increase in mortality rates after decades of decline [1, 2]. The prognosis of CAD differs significantly between genders, with women experiencing higher mortality rates [3, 4]. Women experience more pronounced effects from several risk factors for CAD, including smoking, autoimmune disorders, and psychological stressors, which tend to be either more harmful or more prevalent in women [5, 6]. Studies have further shown that women with angina are less likely to receive invasive diagnostic testing [7]. Nevertheless, when CAD is identified and equitable care is delivered to women and men, both exhibit similar rates of long-term major cardiovascular events [8, 9]. This implies that timely CAD diagnosis in women, followed by prompt initiation of appropriate management, is imperative to bridging the mortality gap between genders.

Given that myocardial ischemia is a time-sensitive condition, diagnostic delays in myocardial infarction have been associated with a two-fold increase in the risk of death [10, 11]. The delay in the diagnosis of CAD in women is often attributed to their unique clinical presentation. Indeed, studies have shown that women with CAD can present with non-specific symptoms like back or abdominal pain rather than the well-defined chest pain more commonly seen in men [12]. This can lead physicians to consider a non-cardiac initial diagnosis and delay primary prevention measures [7, 13, 14].

Angina is the primary symptom in both obstructive and non-obstructive CAD, the latter being more prevalent in women [15, 16]. While guidelines have historically focused on obstructive CAD, non-obstructive causes of CAD can lead to myocardial infarction with non-obstructive coronary arteries (MINOCA), which have comparable clinical outcomes to obstructive MI [17, 18]. Alarmingly, non-obstructive CAD has no standardized diagnostic algorithm, as traditional tests often yield falsely negative results, making anginal symptoms a key diagnostic clue in those patients [19].

Given the importance of early recognition of angina, further research is needed to explore barriers women encounter in obtaining an accurate and timely diagnosis, even when presenting with similar symptoms and demographic characteristics to their male counterparts. The National Health and Nutrition Examination Survey (NHANES) offers a comprehensive and nationally representative platform to analyze potential gender disparities in CAD diagnosis. Utilizing NHANES data, we hypothesized that women would have increased odds of missed angina. This study aims to: 1) explore potential disparities in timely CAD diagnosis, 2) characterize demographic characteristics in patients with missed angina and 3) determine differences in the frequency of associated symptoms with angina between women and men.

## Methods

### Sex versus gender

Although the terms “sex” and “gender” are often used interchangeably, they express different meanings. Sex is defined by a person’s chromosomes or gonads and can be categorized as male, female, and intersex. Gender refers to socially constructed characteristics of women and men and includes roles and behaviors that can differ between cultures. Per the World Health Organization (WHO), gender can influence a person’s experience in healthcare as gender is “hierarchical and produces inequalities that intersect with other social and economic inequalities” [20]. Hence, the term "gender" was used for the purposes of this study.

### Study design and study participants

**Fig. 1:**
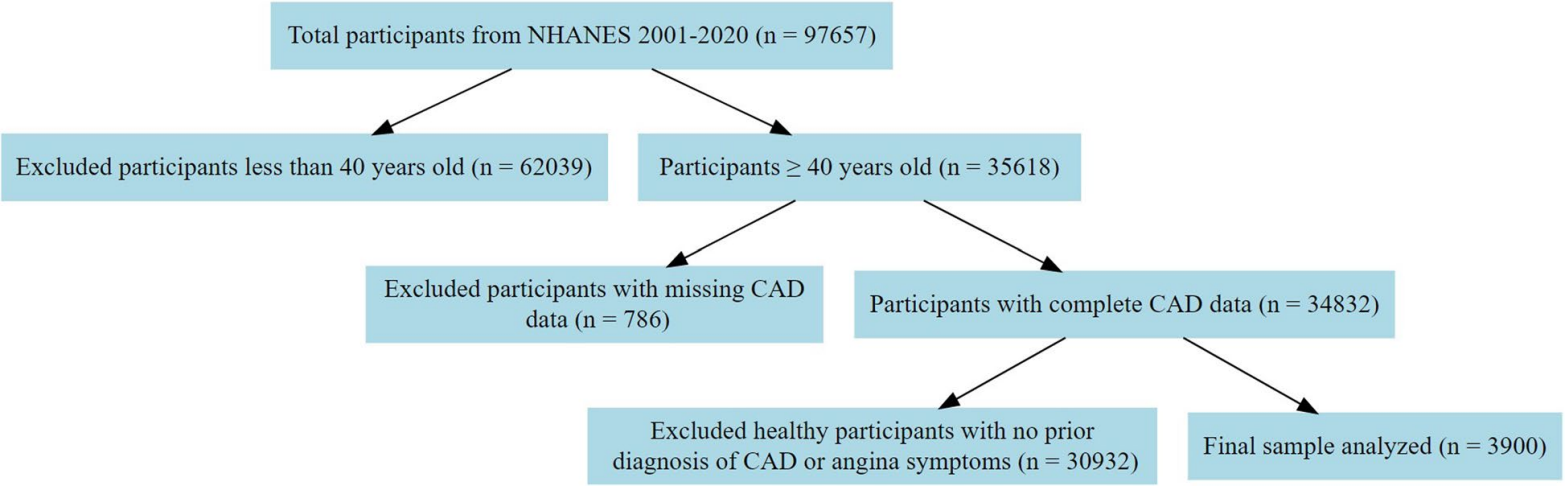
Flowchart for patient selection. Flowchart describing the analytic sample selection for a study investigating the relationship of missed angina to gender using data National Health and Nutrition Examination Survey (NHANES) from 2001 through 2020

We used data from NHANES, which is a stratified multistage survey that combines interviews and physical examinations of non-institutionalized adults and children in the U.S. to assess their health and nutritional status. De-identified data from NHANES are typically released in 2-year cycles. We included nine cycles of NHANES (2001–2002, 2003–2004, 2005–2006, 2007–2008, 2009–2010, 2011–2012, 2013–2014, 2015–2016, 2017–2020). We excluded participants who 1) were younger than 40 years, 2) had unknown CAD diagnosis, and 3) were healthy and did not report either anginal symptoms or a prior CAD/angina pectoris diagnosis. A detailed flowchart for patient selection in the analyzed study sample is shown in Fig. 1.

### Assessment of CAD

We ascertained missed angina using two NHANES questionnaires. First, we used the medical conditions questionnaire to identify participants who answered “No” to either “Has a doctor or other health professional ever told you that you had angina, also called angina pectoris?” or “Has a doctor or other health professional ever told you that you had coronary heart disease?”. Next, we used the Rose Questionnaire, a WHO-developed screening tool used in epidemiological studies to differentiate cardiac from non-cardiac chest pain [21]. The questionnaire has a reported sensitivity of 83% and a specificity of 97% [22, 23]. Participants who screened positive for angina on the Rose Questionnaire but had never been diagnosed with angina pectoris or CAD were categorized as ‘missed angina.’ Those who reported a healthcare professional diagnosis were categorized as ‘diagnosed CAD.’ These two groups were mutually exclusive.

### Mortality

We ascertained mortality by linking participants’ sequence numbers (SEQN) from NHANES to death certificate records from the National Death Index. Mortality follow-up was available through December 31, 2018. Participants from the 2019–2020 NHANES cycle were excluded from mortality analysis due to unavailable follow-up data.

### Covariates

NHANES collects participant data from interviews and examinations conducted at mobile examination centers. The NHANES field office staff checks interview records for accuracy and completeness before they are released publicly [24]. Due to the smaller sample size in some race categories, we combined the races “Mexican American” and “Other Hispanic” into “Hispanic” to ensure reliable estimates. For the poverty level, we used the ratio of family income to poverty (PIR), which was capped by at 5 in NHANES data, even if the ratio exceeded that value, to protect against participants’ identification. Participants who smoked > 100 cigarettes and currently smoke every day or some days were listed as smokers. Daily alcohol consumption was considered present in women with four drinks/day or men with five drinks/day [25]. We defined single status as those who were widowed, divorced, separated, or never married. The diabetes, hyperlipidemia, and hypertension were self-reported.

### Statistical analysis

Table 1 presents participant characteristics stratified by gender and missed angina status. In descriptive analysis, we compared sample demographic characteristics between the diagnosed CAD group and the missed angina group, the Chi-square test was used for categorical data, and the Mann–Whitney test was used for continuous data. Visualization of data spread showed a non-normal distribution, which was confirmed by the Shapiro–Wilk tests. Categorical data were represented as percentages, while continuous data were presented as medians and interquartile ranges (IQR).

**Table 1:** Demographic characteristics of included participants by angina diagnosis and gender

Characteristics	Women			Men		
	**Diagnosed** (*n* = 1,187)	**Missed** (*n* = 551)	***P*-value**	**Diagnosed** (*n* = 1,839)	**Missed** (*n* = 323)	***P*-value**
Age, (IQR)	70.00 (61.00, 80.00)	58.00 (48.00, 68.00)	** < 0.001**	71.00 (62.00, 79.00)	59.00 (51.00, 66.50)	** < 0.001**
Race, *n* (%)			** < 0.001**			** < 0.001**
White	667 (56.2)	219 (39.7)		1186 (64.5)	148 (45.8)	
Black	215 (18.1)	166 (30.1)		227 (12.3)	98 (30.3)	
Hispanic	232 (19.5)	130 (23.6)		291 (15.8)	53 (16.4)	
Other/Multi-racial	73 (6.1)	36 (6.5)		135 (7.3)	24 (7.4)	
Education, *n* (%)			0.758			** < 0.001**
Under 9th grade	199 (16.8)	89 (16.2)		291 (15.9)	56 (17.4)	
9th-11th grade	232 (19.6)	96 (17.4)		262 (14.3)	69 (21.4)	
High School	313 (26.4)	155 (28.1)		399 (21.7)	91 (28.3)	
College	322 (27.2)	150 (27.2)		482 (26.3)	73 (22.7)	
College graduate or above	118 (10.0	61 (11.1		401 (21.9)	33 (10.2)	
BMI (IQR)	29.70 (25.51, 34.50)	32.20 (27.16, 36.80)	** < 0.001**	28.77 (25.71, 32.80)	28.90 (25.39, 33.09)	0.788
Uninsured, *n* (%)	74 (8.4)	79 (17.0)	** < 0.001**	86 (6.0)	55 (21.2)	** < 0.001**
Single, *n* (%)	608 (60.7)	239 (54.7)	**0.037**	451 (30.5)	95 (37.5)	**0.030**
Smoker, *n* (%)	205 (17.3)	129 (23.5)	**0.003**	300 (16.3)	123 (38.1)	** < 0.001**

Table 1 exhibits the characteristics of women with missed angina in contrast to women with diagnosed CAD as well as men with missed angina in contrast to men with diagnosed CAD. Significant associations are in bold (*p*-value < 0.05)

Logistic regression models, using a complete case dataset, were conducted to analyze the association between gender and missed angina, with men as the comparator group: Model 1 as a univariable model, Model 2 as a multivariable model adjusted for age and race, and Model 3 as a multivariable model adjusted for age, race, educational level, body mass index (BMI), smoking, alcohol use, insurance status, poverty level, diabetes, hypertension, hyperlipidemia. Participants with any missing covariate data were automatically excluded from the model. Backward stepwise selection of the variables was implemented until the model with the lowest value of the Akaike Information Criterion (AIC) was achieved. The variance inflation factor (VIF) was used to test for multicollinearity between variables. A value of less than 5 was considered as no significant multicollinearity [26]. To evaluate for potential effect modification, multiplicative interaction terms between gender and individual covariates were incorporated into the regression models.

To test the robustness of our results, we conducted a sensitivity analysis using the full dataset, without excluding participants with missing covariate data. After analyzing the missingness pattern, data were assumed to be missing at random (MAR). We performed multiple imputations using the *MICE* package in R [27], generating five imputations with five iterations each. Pooled estimates were calculated using Rubin’s rule. The imputation model included all covariates from the main model. The regression models, using the imputed dataset, adjusted for confounders in the same way as described for the complete dataset. We used appropriate sampling weights, provided by the National Center for Health Statistics, in all regression models to ensure nationally representative results of the U.S. [28]. We sub-analyzed participants who experienced angina and had a cardiac cause of death after participating in NHANES. All analysis was done in R (version 4.3.1) and utilized the *survey* package to account for the NHANES survey design. Statistical significance was defined as a *p*-value < 0.05.

## Results

### Sample characteristics

We identified 35,618 participants aged or older, of whom 3,900 had prior symptoms or a diagnosis of CAD. Our final cohort included 3,026 participants with a diagnosis of angina pectoris or CAD and 874 participants with missed angina. Women accounted for 63% (551) of the missed angina group, while men accounted for 37.0% (323).

Among women with missed angina, 63% were uninsured and 45% were smokers, compared to 50% and 30%, respectively, among women with diagnosed CAD. Women with missed angina were also more likely to be younger, of ethnic minorities, of higher BMI, and be in a relationship compared to diagnosed women (Table 1).

**Fig. 2:**
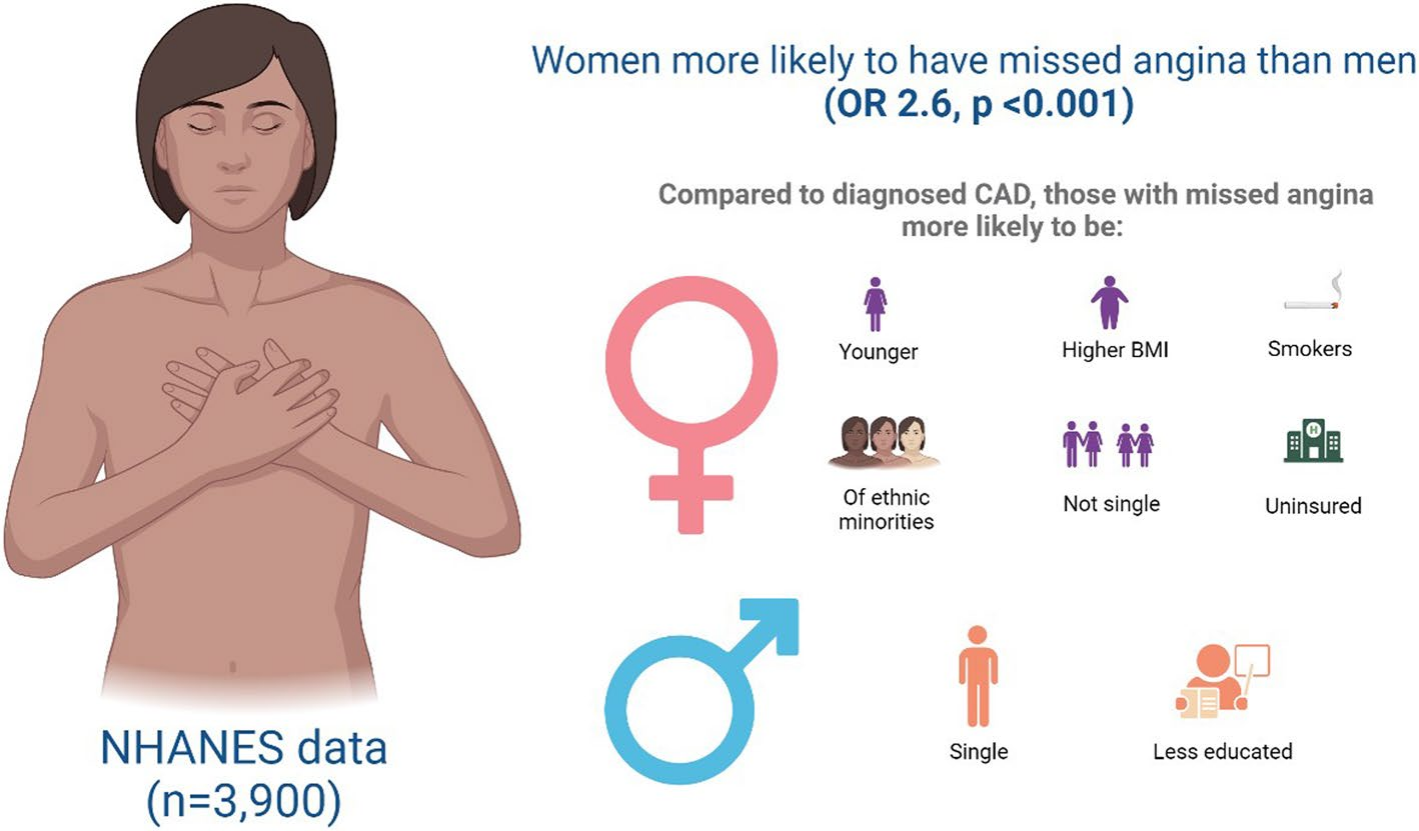
Summary of the main study findings. This figure was created by BioRender.com

A similar pattern was observed in men, except those with missed angina were more likely to be single and less educated. A summary of key demographic differences between genders with missed angina is provided in Fig. 2. Among participants who screened positive for angina, there was no statistically significant difference between women and men in the prevalence of associated symptoms with angina, including dyspnea, epigastric pain, neck pain, right chest pain, and right arm pain (Table 2).

**Table 2:** Associated symptoms in patients who screened positive for angina on the Rose Questionnaire

	**Women (*n* = 727)**	**Men (*n* = 521)**	***P*-value**
Shortness of breath on stairs/inclines, *n* (%)	82 (11.3)	78 (15.0)	0.070
Epigastric pain, *n* (%)	23 (3.2)	17 (3.3)	1.00
Neck pain, *n* (%)	72 (9.9)	42 (8.1)	0.310
Right chest pain, *n* (%)	92 (12.7)	70 (13.4)	0.749
Right arm pain, *n* (%)	43 (5.9)	32 (6.1)	0.963

Table 2 compares the number of women and men in absolute numbers and percentages who experienced associated symptoms with their angina. Significant associations are in bold (*p*-value < 0.05)

### Association between missed angina and gender

**Fig. 3:**
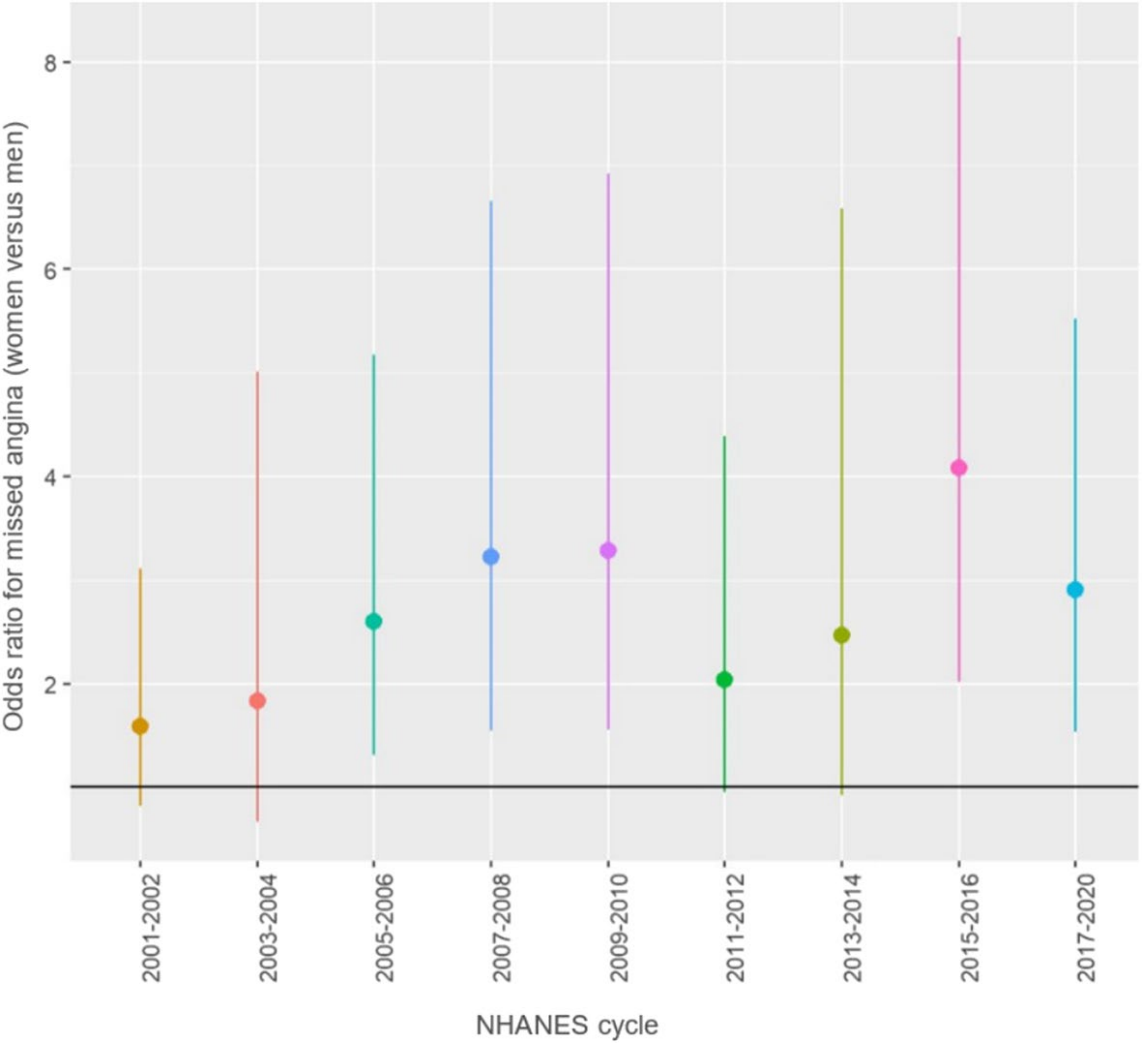
Trends in missed angina odds ratios (women vs. men) across NHANES cycles. This figure illustrates the unadjusted odds ratios and 95% confidence intervals for missed angina in women compared to men across NHANES cycles from 2001–2002 to 2017–2020. Points represent the odds ratios, while vertical lines represent 95% confidence intervals. The line of no effect (odds ratio = 1) is represented by the black horizontal line

Among participants who experienced angina, women had significantly higher odds of missed angina compared to men across all models: model 1 with an odds ratio of 2.62 (95% CI: 2.06, 3.34), model 2 with an odds ratio of 2.61 (95% CI: 2.01, 3.40) and model 3 with an odds ratio of 2.61 (95% CI: 1.73, 3.94) (Table 3). Figure 3 illustrates temporal changes in the model 1 odds ratio across different NHANES cycles.

**Table 3:** Association between missed angina and gender

	**Model 1**		**Model 2**		**Model 3**	
	OR (95% CI)	*P*- value	OR (95% CI)	*P*- value	OR (95% CI)	*P*- value
**All participants**						
Men	*Reference*	*-*	*Reference*	*-*	*Reference*	-
Women	2.62 (2.06, 3.34)	** < 0.001**	2.61 (2.01, 3.40)	** < 0.001**	2.61 (1.73, 3.94)	** < 0.001**
**Participants with a cardiac cause of death at follow-up**						
Men	*Reference*	**-**	*Reference*	**-**	*Reference*	**-**
Women	2.63 (1.16, 5.94)	** < 0.001**	3.39 (1.59, 7.25)	** < 0.001**	3.02^a^ (1.18, 7.75)	** < 0.001**

Table 3 exhibits the association between gender and missed angina across 3 models among all included participants and among the subgroup of participants who had a cardiac cause of death at follow-up. Significant associations are in bold (*p*-value < 0.05)

^a^adjusted for age, race, educational level, BMI, poverty level, smoking, and diabetes

**Fig. 4:**
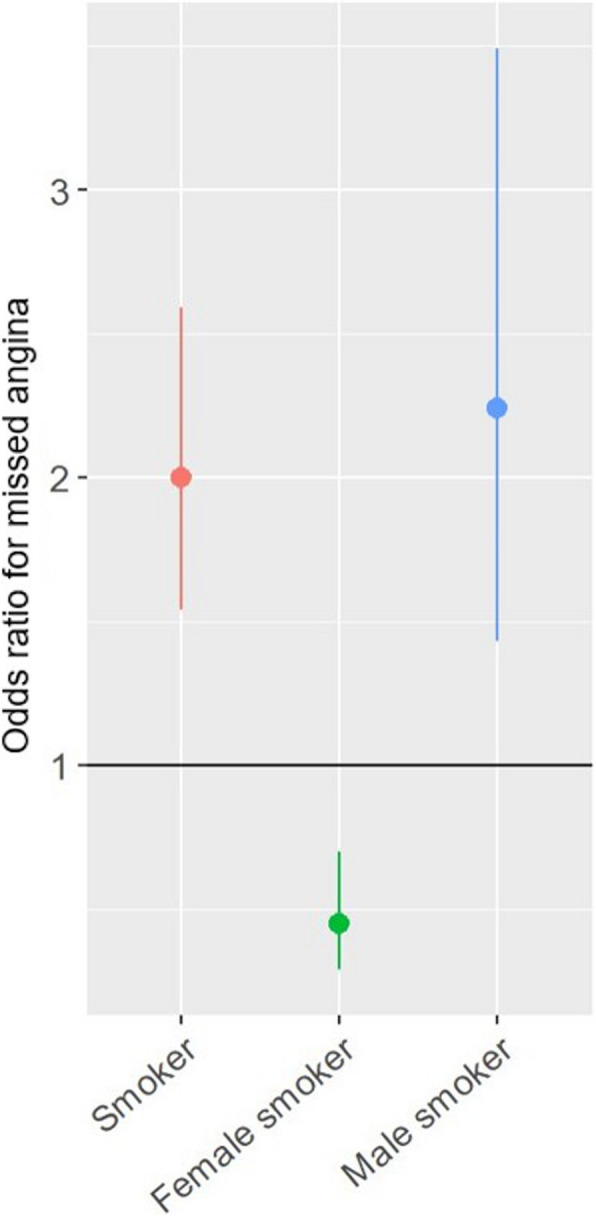
Effect of Smoking on Missed Angina: Odds Ratios by Gender. This figure illustrates the interaction between smoking and the odds of missed angina. Overall, smoking was associated with increased odds of missed angina. However, when stratified by gender, this pattern persisted in men, whereas in women, smoking appeared to be a protective factor against missed angina. that smoking increased the odds of missed angina

Subsequent backward stepwise selection of covariates did not produce a new model as it did not lower the AIC. There was no significant multicollinearity between variables (VIF < 3 in all covariates). Sensitivity analysis, which included patients with missing covariate data through multiple imputations, yielded an odds ratio of 2.62 (95% CI: 2.07, 3.33,* P*** < **0.001) for model 1, 2.61 (95% CI: 2.01, 3.39,* P* < 0.001) for model 2, and 2.40 (95% CI: 1.83, 3.15,* P* < 0.001) for model 3. Interactions between gender and covariates were either insignificant or did not persist after adjusting for other covariates, except for the interaction between women and smoking. Women who smoke were at lower risk for missed angina across all models: model 1 with an odds ratio of 0.45 (95% CI: 0.29, 0.70, *P* < 0.001), model 2 with an odds ratio of 0.42 (95% CI: 0.29, 0.70, *P* = 0.001), and model 3 with an odds ratio of 0.37 (95% CI: 0.19, 0.71,* P* = 0.003). Figure 4 illustrates the interaction between smoking and gender in missed angina odds.

Of the 3,900 participants, mortality follow-up was available for 1,403 participants (560 women and 843 men). A total of 444 participants (175 women and 269 men) died due to a cardiac cause. Women were significantly more likely than men to have screened positive for angina on the Rose Questionnaire before their death (28.70% versus 18.40%,* P* = 0.02). In the subgroup who experienced angina and later died from cardiac causes, missed angina remained more likely in women compared to men with an odds ratio of 2.63 (95% CI: 1.16, 5.94) in model 1, 3.39 (95% CI: 1.59, 7.25) in model 2, and 3.02 (95% CI: 1.18, 7.75) in model 3. Further analysis of deceased women in this subgroup showed that, compared to women alive at follow-up, deceased women were older (75.55 ± 8.73 versus 72.18 ± 10.94, *P* < 0.001) and more likely to be white (76.6% versus 64.7%, *P* = 0.031), but were significantly less likely to be smokers (10.3% versus 17.4% *P* = 0.041). No significant differences were observed in the subgroup analysis between men alive versus deceased at follow-up.

## Discussion

Our study spanning 20 years of NHANES data found that women who screened positive for angina were significantly less likely to have received a prior diagnosis of CAD than men. This underdiagnosis is not merely a byproduct of women being less likely to have CAD as the cause of their chest pain (and therefore of physicians correctly diagnosing a non-CAD cause of chest pain), as it also extends to women who later ended up dying of cardiac causes. These findings persisted after accounting for baseline characteristics. Our findings align with previous studies showing that cardiovascular disease is more likely to go undiagnosed in women [29–32].

Disparities in timely CAD diagnosis are often attributed to different clinical presentations of myocardial ischemia [33–37]. However, questions in the Rose Questionnaire are not modified based on the gender of the participant. The questionnaire is designed to primarily identify classic anginal symptoms and has lower reported sensitivity in women [38]. Nevertheless, women were still observed to have greater cases of missed angina. This suggests that the true effect estimate is greater than that observed in our study, as more false negatives in women would lead to fewer women being correctly categorized as having missed angina.

Notably, both genders with CAD experience a significant overlap of symptoms [39, 40]. A prospective study on MI patients presenting to the emergency department (ED) found that 92% of women presented with chest pain, compared to 91% of men (*P* = 0.439) [41]. However, women reported more associated symptoms with their chest pain [32, 42]. Indeed, Ferry et al. found that women with MI were more likely to report experiencing nausea and pain radiating to the neck and jaw alongside chest pain, which could be why some retrospective studies labeled the presentation of angina in women “atypical” [41]. Of note, our study did not find a significant difference in the frequency of associated symptoms with angina between women and men.

Since there was no difference in the clinical presentation of CAD, in terms of both the character of chest pain and the frequency of associated symptoms, we speculate that the reason for missed angina in women lies beyond their symptoms. ‘Knowledge-mediated’ gender bias occurs when physicians dismiss symptoms of a disease that is less common in the patient’s gender [43]. Women who do have a cardiac cause of chest pain are faced with the exaggerated notion that ischemic heart disease is primarily a male disorder [31, 44]. In a cohort of 672 patients with chest pain, the prevalence of a cardiovascular etiology was only 2.7% lower in women (14.8% vs 17.5%). Despite that, women with chest pain were less than half as likely to be referred to a cardiologist (7.4% vs 16.6%) [44].

On the other hand, physicians who evaluate women for suspected CAD must rely on studies that primarily enrolled male participants to guide their workup [45]. For instance, the widely used cut-off values for cardiac troponin may lead to a significant number of missed diagnoses in women as their troponin lags ~ 2 decades behind age-matched men [46]. In patients with unstable angina, 43% of men had measurable troponin compared to 27% of women [47]. Moreover, classic imaging techniques, such as invasive and CT angiography, are effective in detecting epicardial coronary stenosis—more common in men—but struggle to identify non-obstructive types of CAD, like coronary microvascular disease, which is more prevalent in women [5, 19].

In many instances, however, the patient herself misses her symptoms. Patients who cannot recognize that they are experiencing cardiac chest pain are more likely to delay seeking medical care [48]. The increased pre-hospital delay in women with chest pain compared to men is well-documented, with trend analysis showing that women have had a smaller decline in their pre-hospital mortality [49–52]. When interviewing women post-hospitalization in the coronary care unit, women reported they had difficulty linking their symptoms to CAD and waited at home for symptoms to disappear even when their pain became severe [53].

It is worth noting that patients with missed angina in our study had lower rates of health insurance, raising the question of whether fear of prohibitive healthcare costs could play a role in a patient’s decision to stay home. Proximity to care could have also been a contributing factor. While distance to the nearest healthcare facility was not reported in NHANES, it is a well-documented barrier to healthcare access, particularly in rural areas. Despite the US being largely rural, most physicians, particularly specialists, practice in urban areas due to service demand from the higher population density [54]. Notably, the exertional dyspnea common in CAD patients may have made accessing remote healthcare facilities particularly challenging for them compared to other patient populations.

Hence, it is worthwhile to consider alternative mechanisms behind the strong association between women and missed angina in our study. For instance, women experiencing angina and not having access to healthcare – due to lack of insurance or remote location– could have been more likely to agree to participate in the NHANES survey, which offers a free check-up at the participant’s home and the mobile testing center. On the other hand, men with undiagnosed CAD are less likely than women to experience angina, as established in the present study and in the literature [55]. The lack of angina in men with undiagnosed CAD could have decreased their motivation to participate in NHAES, causing women with missed angina to be overrepresented in our sample.

One factor potentially underlying women’s decision to forego medical care is an underestimation of their cardiovascular risk [56, 57]. The 2019 American Heart Association (AHA) National Survey showed that the proportion of women aware that heart disease is their most likely cause of death had decreased compared to the 2009 survey (43.7% vs. 64.8%, *P* < 0.05) [58]. This could be partially attributed to another disparity women experience in healthcare, as only 22% of primary care physicians reported feeling “extremely well-prepared” to assess cardiovascular disease risk in women [59]. We speculate that physicians are less likely to counsel their patients on their cardiovascular risk if they do not perceive said patients to have an elevated risk.

In our study, women who smoked were at lower odds for missed angina. We hypothesize that though smoking increased the risk of CAD in women, it also increased their odds of diagnosis as it served as an alarm signal for physicians, decreasing their threshold for referral when presented with a woman who smokes complaining of chest pain. This may be influenced by the extensive literature reporting on the disproportionality harmful impact of smoking on women [60, 61]. Indeed, more than two-thirds of internists in the U.S. are aware that tobacco use is the number one cause of MI in women younger than 50 years old [62]. While this risk-aware assessment of women who smoke may reduce missed diagnoses, it raises concerns that women without traditional risk factors might not receive the same level of diagnostic scrutiny.

This finding could also be due to women who smoke being more likely to seek medical attention if they develop chest pain. A U.S. multicenter study found that smokers were more likely than non-smokers to identify smoking as a risk factor for cardiovascular disease [63]. Indeed, knowledge of the adverse effects of smoking has become wildly accessible due to well-funded mass media public health campaigns, aimed at smokers or those at risk for smoking [64]. Data from the Health Information National Trends Survey further showed that women were more likely than men to reject smoking myths, such as believing that exercise or vitamins can undo most of the effects of smoking [65]. Notably, no significant interaction was observed between women and other risk factors for CAD – like hypertension or diabetes. However, we acknowledge that the relationship between smoking and missed angina is still speculative and should be interpreted with caution as we could not rule out residual confounding or recall bias skewing the results.

Nevertheless, this relationship could signal that when targeted public health movements are implemented, patients respond to a call to action. Indeed, after participating in the Go Red for Women event by the AHA, a movement aiming to raise awareness about cardiovascular disease in women, 70% of women went for health screening [66]. Yet, the annual allocation for the Prevention and Public Health Fund (PPHF) is currently less than half of what Congress had announced, due to repeated funding cuts [67]. The PPHF funds crucial movements in women’s health like the Well-Integrated Screening and Evaluation for Women Across the Nation (WISEWOMAN) program, which targets uninsured women between the ages of 40 to 64 for cardiovascular screening [68] – the same demographic observed in our study to have missed angina.

Our findings have crucial implications for timely CAD diagnosis in women. It is evident that to rectify gender disparities policies from governmental bodies ensuring women’s access to preventive health programs need to be upheld. Increased investments in community-based programs and mobile health units targeting marginalized populations are crucial to ensure women receive timely cardiovascular risk assessments and preventive care. Moreover, focused efforts from researchers and academic journals are key to refining our understanding of how CAD presents in women and developing gender-specific risk assessment tools. Equal participation of women should be a criterion for cardiovascular clinical trials to be eligible for funding initiatives. To further counteract the gender disparity, preclinical studies should adhere to the National Institutes of Health’s Sex as a Biological Variable (SABV) research policy requiring researchers to not exclude female subjects (cells/tissues/animals) from their work [69]. Future research should address the feasibility of modifying in-use CAD risk calculators to include additional risk factors specific to women, like types of birth control or hormone replacement therapy used, conditions related to pregnancy, and polycystic ovarian syndrome [70]. To build on the findings of this study, large-scale observational studies applying confirmatory tests for CAD after initial screening are needed to affirm the true effect estimate of the gender disparity.

The results of our study should be interpreted within the context of its limitations. First, the healthcare records of the participants in our study are unavailable in NHANES, hence, there is no way to verify their self-reported diagnoses or lack thereof. While self-reported data poses a risk of recall bias, non-differential misclassification typically bias estimates towards the null [71]. Second, the Rose Questionnaire was initially developed and validated in men, with reports of the questionnaire having higher specificity for CAD in men than in women. To increase the positive predictive value of this screening tool, we performed a subgroup analysis that included only participants who died due to cardiac causes after screening positive for angina on the questionnaire. Third, in participants with available follow-up mortality data, we could not ascertain the precise cause of cardiac death, although coronary disease is by far the most common [72]. Lastly, plausible confounders such as proximity to care could not be accounted for as they were not reported in NHANES.

## Conclusion

Our study found a persistent disparity of timely angina diagnosis among women, even when their clinical presentation and demographic characteristics were indistinguishable from men’s. This relationship extended to individuals who ultimately died of cardiac causes. Gender bias is suspected to be a strong factor behind this relationship. Preventive health programs and campaigns, particularly those targeting disadvantaged women, could counteract this disparity. Future studies adjusting for proximity to care and performing confirmatory tests on patients with angina are needed to estimate the true effect estimate of the disparity women face. Addressing this gap is imperative to reducing preventable mortality in women.

## Data Availability

The datasets generated and analyzed during the current study are available in the NHANES database, www.cdc.gov/nchs/nhanes.
